# An Innovative Study for Tool Wear Prediction Based on Stacked Sparse Autoencoder and Ensemble Learning Strategy

**DOI:** 10.3390/s25082391

**Published:** 2025-04-09

**Authors:** Zhaopeng He, Tielin Shi, Xu Chen

**Affiliations:** 1 School of Mechanical Science and Engineering, Huazhong University of Science and Technology, Wuhan 430074, China; hezhaopeng1007@163.com; 2 School of Energy and Power Engineering, Huazhong University of Science and Technology, Wuhan 430074, China; chenxu@dfcv.com.cn

**Keywords:** tool wear prediction, ensemble learning, deep learning, autoencoder, multi-sensor fusion, milling

## Abstract

**Highlights:**

**What are the main findings?**

- The SSAE-BPNN models can be trained as multiple deep learning models with different predictive performance by using different activation function configurations of the hidden layer.
- The SSAE model can learn the deep fusion features that reflect the change of tool wear from the combination of multi-sensor sensitive features.

**What is the implication of the main finding?**

- The integrated learning model based on the stacking learning strategy used the SSE-BPNN models with different prediction performance as the primary learners and the Bayesian optimized GBD model as the secondary learner to construct the intergrated deep learning model for tool wear prediction.
- The proposed model further improved the predictive performance of tool wear prediction model based on deep learning method.

**Abstract:**

Accurately predicting tool wear in real time is crucial to enhance the tool prognostics and health monitoring system in computerized numerical control (CNC) machining. This paper proposed a novel integrated deep learning model for predicting the wear of milling tools by fusing multi-sensor features. The raw signals of vibration and cutting force acquired from the continuous cutting cycle were used to extract multi-sensor features throughout the full lifecycle of the milling tools in time, frequency, and wavelet domains, respectively. The sensitive features from these signals were identified through correlation analysis and used as input for the stacked sparse autoencoder (SSAE) model with backpropagation neural network (BPNN) as the regression layer to predict tool wear. SSAE models with different activation function configurations of hidden layers were utilized to construct deep neural network models with different prediction performance, which were taken as primary learners of integrated deep learning model. The intergrated SSAE model based on the stacking learning strategy applied the gradient boosting decision tree (GBDT) regression model with Bayes optimized hyperparameters as the secondary learner to predict tool wear. Compared to the single SSAE model and shallow machine learning models, the proposed method significantly improved both the prediction accuracy and reliability.

## 1. Introduction

With the first introduction of the concept of Industry 4.0 and the rapid development of the manufacturing industry, intelligent manufacturing has emerged as a key driver in advancing the fourth industrial revolution, which uses information technology to promote industrial transformation and aims to improve the intelligent level of manufacturing. The central goal of intelligent manufacturing is to reduce production costs, improve manufacturing efficiency, and product quality [1]. The machine tool plays the role of the “industrial mother machine” and the machine that produces the machine in the manufacturing industry. Intelligent machine tools combine advanced manufacturing technologies with the latest information technology to realize the self-sensing, self-learning, and self-decision ability, which are the critical equipment of intelligent manufacturing implementation. As direct executor of cutting process such as turning, milling, drilling and so on, cutting tools of intelligent machine tool have always been a crucial factor for machining quality and manufacturing costs. Online monitoring tool status and diagnosing tool wear, breakage or other damage faults are of far-reaching significance for extending tool life, saving processing auxiliary time and improving production efficiency [2]. Tool condition monitoring research has established a mature research framework from sensor signal monitoring to tool condition identification and final decision-making. During the tool cutting process of the material, the phenomenon of tool wear and even breakage directly leads to changes in characteristic information such as cutting force, cutting temperature, vibration, chip color and cutting chatter [3]. Due to factors such as cutting conditions, workpiece materials, and external environment, the tool condition monitoring system becomes highly complex and makes it challenging to ensure accuracy. With the development of intelligent manufacturing technology, the intelligent processing requirements are getting higher and higher. The application of the new generation information technology in tool condition monitoring has been extensively researched to strengthen the robustness and accuracy of intelligent monitoring, which was also the main research trend of intelligent tool condition monitoring.

The prognostics health management (PHM) system comprehensively utilizes the advanced sensing technology and the new generation of information technology to achieve intelligent maintenance and fault pre-diagnosis of mechanical equipment. Model-based and data-driven methods are the two main approaches used for the modeling techniques for cutting systems. The focus of model-based pre-diagnosis is on thoroughly analyzing the system’s mathematical and physical models with the detailed examination of its physical and dynamic processes. However, in most cases, the complexity of mechanical transfer systems make it difficult to obtain the prior knowledge of system behavior. Therefore, data-driven models for indirect realization of tool condition pre-diagnosis based on feature learning of experimental data have been proposed and extensively studied. The development of artificial intelligence technology based on the black box model provides a more effective and accurate modeling method to pre-diagnose tool condition, which is more suitable for the study of highly random and nonlinear cutting signals. Commonly used intelligent pre-diagnosis models based on data-driven include support vector machine (SVM), long-short term memory (LSTM) network, artificial neural network (ANN) and so on, which were widely applied in mechanical equipment intelligent operation and maintenance system. The technical links of tool condition monitoring research are mainly composed of three parts: the selection of monitoring sensor signals, feature extraction and optimization based on signal preprocessing, and pattern recognition or prediction model establishment [4]. Ref. [5] studied data-driven model to monitor tool wear condition based on empirical model decomposition and BPNN model from vibration, force, and acoustic emission signals. Acceleration of mechanical vibrations and cutting forces signals was utilized for tool wear process monitoring. The effectiveness of ANN and multi-layer perceptron for tool wear prediction has been evaluated [6]. Research has been conducted on the relationship between chip surface color and tool wear to develop the predictive model using BPNN and changes in chip color [7]. Compressed sensing technology can be used for data compression and high-precision reconstruction of monitoring sensor signals. By extracting energy parameters from the compressed domain of acoustic emission signals, a comprehensive evaluation index for bearing damage status was established [8]. Sound signal during tool wear process has been discussed as monitoring signals for accurate tool wear prediction using time-frequency feature analysis [9]. Sound pressure and machining force during the milling process were measured, and artificial intelligence techniques presented accurate modeling capability for tool condition evaluation based on the acquired signals [10]. Neighborhood preserving embedding was conducted to fuse the extracted raw cutting force signals features and realize dimension reduction, and integrated SVM has been presented for accurate estimation of the end milling tool wear [11]. The cutting force signals were decomposed into multiple sub-band by wavelet packet decomposition. The optimized least square SVM was trained for tool condition monitoring with the extracted wavelet packet coefficients as the monitoring features [12]. Ref. [13] established the comprehensive tool remaining service life prediction model through support vector regression (SVR) and trajectory similarity. Relevance vector machine has been studied for tool wear prediction to show its advantages, compared with the traditional predictive models such as ANN and SVM [14]. A novel tool wear evaluation method was proposed using integrated radial basis neural network and kernel principal component analysis to accurately monitor tool wear during machining in real time [15]. An optimized gaussian process regression (GPR) model was introduced for forecasting the residual lifespan of low-speed bearings utilizing degradation assessment from monitored acoustic emission signals [16]. A hybrid information system based on LSTM network was presented for tool wear monitoring [17]. The deep bidirectional LSTM network was developed to use sensor data to track tool conditions by constructing time windows so as to predict remaining tool life using limited data [18]. Ref. [19] studied the singular value decomposition for cutting force signals to evaluate the tool wear using bidirectional LSTM neural network. In order to enhance sensor information in various processes, the use of the multi-sensor fusion method was essential to promote the adequacy of monitoring data and reduce uncertainty considering that the dynamic change of the sensor signal was directly affected by the processing status such as cutting parameters, tool condition, workpiece material characteristics, and processing environment. The selection of multi-sensor signal types and fusion method was the critical point to realize the multi-sensor fusion technology, which can obtain more valuable feature information for monitoring tool condition. The current research on obtaining the effective features from multi-sensor signals has received more and more attention [20]. A global multi-sensor fusion feature extraction method was studied for tool condition evaluation in milling process [21]. The acoustic emission and cutting force fusion signals were reduced dimensionally by linear discriminant analysis and an effective model for predicting tool wear was established through the fusion of features using SVM [22]. The model combined acoustic emission, vibration, and cutting force signals collected during dry turning to ensure accurate prediction of tool wear [23]. Ref. [24] presented a new probabilistic nuclear factor analysis, which was used to optimize the fusion of multi-sensor signal characteristics and effectively improved the tool condition monitoring accuracy. Ref. [25] monitored the spindle drive current and acceleration signals to evaluate the predictability of tool wear in the milling process. However, the performance of the model mentioned above relied heavily on manual feature extraction, which was very difficult to process raw sensor data containing a large amount of feature information. In addition, these methods were basically research under experimental conditions, not in a real manufacturing environment.

Due to the swift evolution of the next-generation information technology, data have become more and more inflated and rapidly become larger. There are more and more ways for humans to obtain data. The concept of “big data” has been concerned with large data volume, complex structure, and diverse types. Therefore, the processing of big data faces many challenges and traditional signal processing cannot dig out effective features from large amounts of data. The proposal and extensive research of deep learning methods make people see hope. The study of learning signal features from big data based on deep learning theory can obtain more complete and in-depth feature information than manual feature extraction methods. The deep learning model outperformed shallow machine learning models in extracting deep features from the original data, which provided a solution for the feature learning of complex nonlinear high-dimensional signals. As a result, the application of deep learning for deep feature extraction from monitoring signals in intelligent fault diagnosis and condition monitoring has received widespread attention. A trustworthy tool condition forecasting strategy during processing was implemented using deep learning [26]. The intelligent monitoring system based on deep learning theory made full use of monitoring information and conducting research on online intelligent evaluation and optimization of cutting conditions through processing dynamics analysis and heterogeneous big data fusion [27]. Considering the requirements of low response delay and high precision, Ref. [28] realized online monitoring of tool condition through deep learning and fog calculation methods. The research introduced techniques for classifying tool wear using big data analysis, deep learning, and signal imaging [29]. A new method for predicting tool wear was proposed incorporating deep convolutional neural networks and fusion of multi-domain features [30]. Ref. [31] collected multiple sensor signals in the tool wear process and compared the predictive performance of deep belief network and artificial intelligence methods, such as SVR and ANN, which confirmed the superiority of the deep belief network in predictive performance. Therefore, deep learning techniques were capable of learning intricate features from various sensor signals and establishing more accurate tool wear prediction models, which is worth continued studied.

This paper proposed an integrated SSAE model that learned deep fusion features from multi-sensor data to reflect tool wear changes and improve model prediction accuracy through ensemble learning method, which is a novel study for tool wear prediction with integrated deep learning theory combined with multi-sensor fusion technology. PHM 2010 challenge datasets included acoustic emission, vibration, and cutting force signals along three axes recorded during the complete cutting process of milling tool. Each data sample matched one cutting cycle with the micromeasurement of the wear on the three edges of the milling tool as calibration values. Firstly, feature extraction and correlation analysis were applied to select sensitive features combination from multi-sensor signals. Secondly, multiple single SSAE models with different neuronal activation function configurations were trained to establish deep neural network models with different tool wear prediction performance. The modified loss function in the training process was available for promotion of the generalization ability and robustness of the learned deep reconstruction features. Finally, the proposed model based on the stacking learning strategy utilized the multiple single SSAE models as the primary learner and shallow machine learning regression model as the secondary learner to establish the tool wear prediction model.

The remainder of this paper is organized as follows. The theoretical background of SSAE model are explained and the network architecture of the proposed tool wear prediction model are briefly described in Section 2. Description of PHM 2010 tool wear datasets and details of experiment setup are illustrated in Section 3. The comparative analysis of different predictive models is discussed in Section 4. Finally, the research conclusions of this study are summarized in Section 5.

## 2. Architecture of the Proposed Method

### 2.1. Stacked Sparse Autoencoder

**Figure 1:**
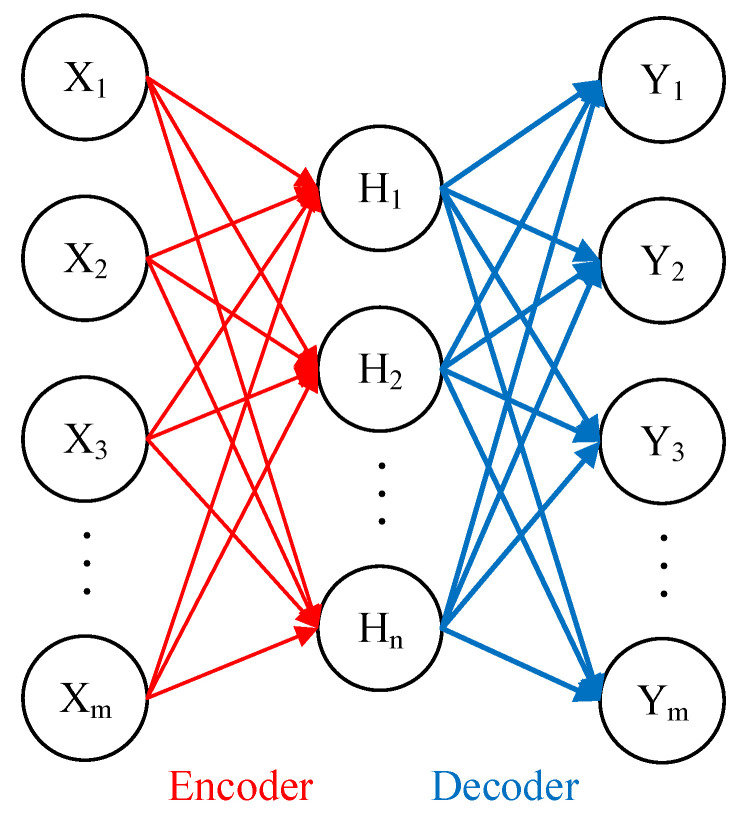
The diagram of network connection for the standard AE mode.

The network topology for the standard autoencoder (AE) model consists of the three-layer neural network as shown in Figure 1. Additionally, both the input and output layers contain the same number of neurons. The transformation of the input vector to the hidden layer feature output is the encoding process of the AE model, while the decoding process is the transformation of the hidden layer feature output to the output layer. The training of the AE model focuses on minimizing the loss function with the goal of reconstructing the input data from the output data. Hidden layer features can be used as input data reconstruction and deep feature expression. The inclusion of the sparsity term in the loss function employed in training the AE model leads to the creation of the sparse AE model, so that the output of hidden layer features has sparsity. The sparse AE model can learn abstract features and structure of the input data more effectively with the mathematical calculation formula of the training process defined as follows:

(1) H=XW+b

(2) $$Y=HW^{\prime }+b^{\prime }$$

(3) $$KL(\rho \left\|{\hat{\rho }}_{i}\right.)=\rho \log\frac{\rho }{{\hat{\rho }}_{i}}+(1-\rho )\log\frac{\left(1-\rho \right)}{\left(1-{\hat{\rho }}_{i}\right)}$$

(4) $$L_{loss}=\frac{\sum_{i=1}^{N}\left(X_{i}-Y_{i}\right)}{N}+\beta \sum_{i=1}^{n}KL(\rho \left\|{\hat{\rho }}_{i}\right.)+\frac{\lambda }{2}\sum_{i=1}^{m}\sum_{j=1}^{n}W_{ij}^{2}$$

Where m and n representing the number of neurons in the input and hidden layers with N denoting the number of training samples in the sparse AE model. X, Y, and H are used as the input data, the output data, and the feature output vectors of the hidden layer, respectively. W, b, W′, and B′ are the connection weight parameters and bias matrices propagated forward by the input, hidden, and output layers. L_loss_ indicates the loss function in the training process, which is composed of reconstruction error, weight regularization term. and sparse penalty term. β and λ are sparse term factor and weighted regular term factor. KL (ρ||${\hat{\rho }}_{i}$) in the loss function is the Kullback–Leibler (KL) divergence as an additional penalty factor to restrict the sparsity of the activation outputs of the neurons in the hidden layer. ${\hat{\rho }}_{i}$ and ρ are the average activation value of the i-th hidden layer neuron and the sparse coefficients.

**Figure 2:**
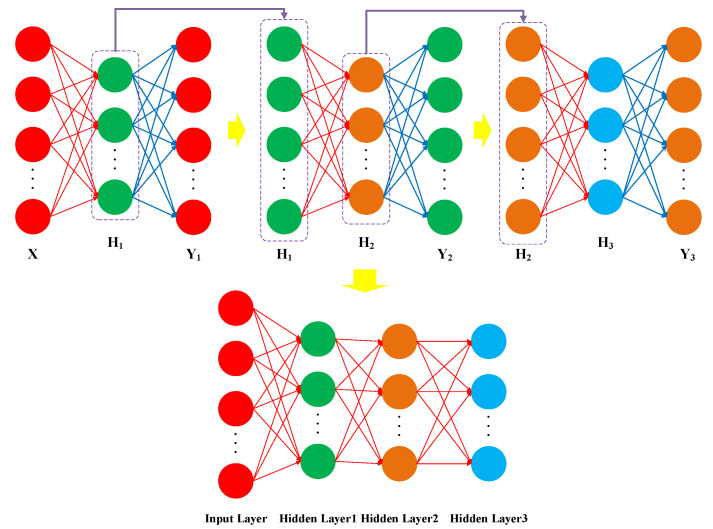
The network structure and training process of SSAE model.

The step-by-step training and stacking of hidden layers from multiple sparse AE models ultimately construct the SSAE model. The network structure and training process of SSAE model are exhibited in Figure 2. Upon completing the training process with the minimum loss function, the hidden layer outputs that reflect the reconstructed input data features are generated from the first sparse AE model, which are subsequently fed as the input vector to the second sparse AE model for training in the same manner. After the sequential training of multiple sparse AE models, the encoder connection weights of each sparse AE model are applied as the connection weight between the two neural network layers corresponding to the SSAE model. After training the SSAE model, the original input signal features are mapped to the output representations from the top hidden layer to achieve deep feature learning. Finally, the multi-layer neural network-based SSAE model is constructed.

### 2.2. The Structural Framework of the Proposed Method

**Figure 3:**
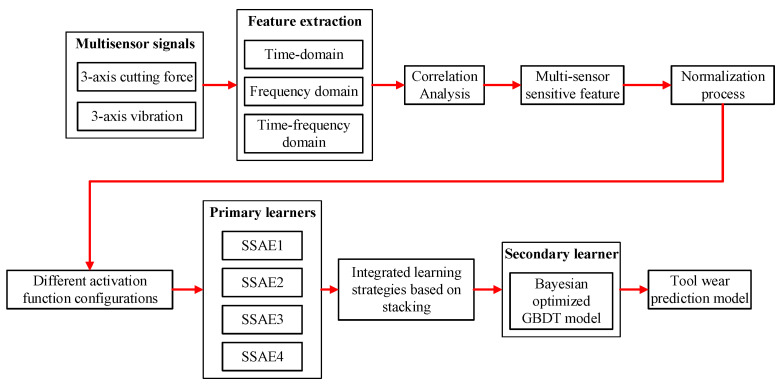
The construction and calculation processes of the proposed model.

Deep learning method can learn deeper feature information from monitoring sensor signals with more powerful feature learning ability as opposed to shallow machine learning models, which makes it an important research field to improve the indirect monitoring accuracy of tool condition. Multi-sensor signal fusion technology can provide more abundant and comprehensive tool condition monitoring information to improve the monitoring effect. SSAE as an unsupervised deep learning model performed deep fusion feature learning on input multi-sensor signals with BPNN as the regression layer. The three-axial cutting force and vibration signals monitored online in milling tool wear experiment were extracted and optimized in time, frequency, and wavelet domains, respectively. The combinations of sensitive features selected by correlation analysis were normalized and put into the SSAE-BPNN model, thereby constructing the deep neural network prediction model for tool wear. The SSAE model can construct tool wear prediction models with different prediction performance through different activation function configurations of hidden layers. The intergrated deep learning model with stacking learning strategy used the multiple single SSAE models with different prediction performance as the primary learners with the hyperparameter optimized GBDT regression model as the secondary learner. Finally, the construction and calculation processes of the proposed model with multi-sensor signals input and ensemble learning strategy are shown in Figure 3.

## 3. Milling Experiment and Data Acquisition

### 3.1. Milling Wear Experiment

**Figure 4:**
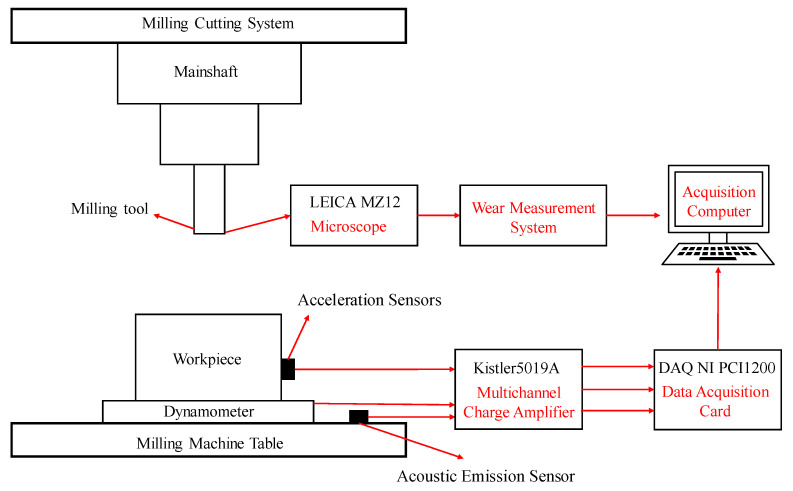
The schematic diagram of milling experiment platform and sensor installation layout.

The predictive accuracy of the proposed model was experimentally evaluated using the milling wear datasets from the PHM 2010 tool health prediction competition. Wear experiment of the milling tool was realized by continuous cutting of the workpiece surface through dry milling on CNC milling machine (Roeders Co. Ltd., Soltau, Germany). The schematic diagram of the milling experiment platform, multi-sensor installation, and acquisition system is shown in Figure 4. The experimental conditions in high-speed milling process are listed in Table 1. Wear experiment was repeated for six full life cycle tests under the above cutting conditions. Square stainless steel (HRC52) workpieces were used as end milling materials and the CNC milling machine used a pre-defined tool path with cutting parameters set at 10,400 rpm spindle speed, 1555 mm/min feed rate, 0.125 mm radial cut depth, and 0.2 mm axial cut depth to remove raw material from the workpiece surface. The three-directional vibration data and acoustic emission signals were monitored from Kistler piezoelectric accelerometers (Kistler Group, Winterthur, Switzerland) mounted on the workpiece and Kistler acoustic emission sensor (Kistler Group, Winterthur, Switzerland) positioned on the workbench to transmit the collected signals in the form of voltage signals. The three-axis cutting forces signals during milling were acquired using the Kistler piezoelectric dynamometer (Kistler Group, Winterthur, Switzerland) positioned between the milling machine table and the workpiece to convert these signals into voltage data. The Kistler 5019A multichannel charge amplifier (Kistler Group, Winterthur, Switzerland) amplified the original voltage signals from all the sensors and these signals after processing were subsequently fed into the NI DAQ PCI 1200 data acquisition card to facilitate real-time acquisition of the multi-sensor signals with 50 kHz sampling frequency.

### 3.2. Description of the Datasets

The PHM 2010 datasets recorded the three-axial cutting force, three-axial vibration, and acoustic emission signals in the milling wear experiment. Six three-flute ball nose tungsten carbide cutters were subjected to wear experiments with each cutter executing approximately 315 cutting cycles under the same working conditions mentioned above with collected datasets named C1–C6 in turn. LEICA MZ12 high-performance stereomicroscope (Wetzlar, Germany) was used for offline measurement of the microscopic wear status of the three cutting blades on the milling cutter after the end of each face milling cycle. The wear measurement labels for multi-sensor monitoring signals were collected after each cutting cycle for the C1, C4, and C6 datasets. Simultaneously, these three datasets were selected as experimental datasets to verify the tool wear prediction performance of the proposed model in this paper.

## 4. Results and Discussion

Features extraction was conducted for the multi-sensor signals monitored in milling tool wear experiments from time, frequency, and wavelet domains. The calculated features from multiple domains consisted of 17 time-domain features such as peak, mean, minimum, variance, efficient of variation and so on, 7 frequency-domain features based on Fourier transform such as average amplitude, center frequency and so on, and 8 wavelet coefficients energy feature based on three-layer wavelet packet decomposition with Meyer wavelet as the wavelet basis extracted from 50,000 raw data points, which were selected from the acquired data of each cutting cycle as sample data. The detailed calculation formulas for these 32 features are provided in [32]. Considering that the X-axis cutting force signal was found to have both deviation and error in the C1 dataset, the remaining six columns of sensor signals were subjected to multi-domain features extraction. Additionally, the average wear on the three sharp edges of the milling cutter was measured and calibrated for each cutting cycle to reflect the actual wear. The combination of sensitive features was determined by the association between the extracted features and the variations in measured wear throughout the entire lifecycle of milling tool as calculated by the Pearson correlation coefficient.

The combinations of selected sensitive features were normalized and provided as initial input data of the SSAE model. The BPNN model was utilized by establishing the regression prediction model between the output features of the highest hidden layer and the actual tool wear value, thus constructing the deep neural network model. Finally, the model’s prediction performance was further improved by fine-tuning the parameters through the minimization of the loss function. The hyperparameters requiring optimization in the constructed SSAE model included the count of nodes in the hidden layers and the number of network layers. Simultaneously, other model training parameters were configured using empirical parameters including learning_rate, batch_size, and epochs. In the AE model, the optimal node count in the hidden layer was selected based on minimizing the reconstruction error and the prediction error of tool wear for the constructed deep neural network model was minimized to evaluate the number of network layers in the SSAE model. The sensitive feature combinations with absolute Pearson correlation coefficients greater than 0.6, 0.7, and 0.8 were normalized and subsequently input into the SSAE model with sigmoid function as hidden layer activation function to perform the network structure hyperparameter training based on Bayesian optimization. The selection range of the number of hidden layer nodes for each layer in the SSAE model during layer-by-layer training was based on the number of nodes in the previous layer with the compression ratio factor controlled between 1 and 3 for optimization and the number of hidden layers were determined to be between 2 and 4. The RMSE of the SSAE models’ prediction results with final trained different network parameters is listed in Table 2 with the three hidden layers of SSAE model exhibiting optimal prediction performance and the selection of hyperparameters ρ and β based on the Bayesian optimization process with 50 iterations. The SSAE model with correlation coefficient of sensitive features greater than 0.8 and the number of neuronal nodes of 58-43-25-15 had the best prediction accuracy for tool wear, which was selected as the hyperparameter configuration for single SSAE model.

**Figure 5:**
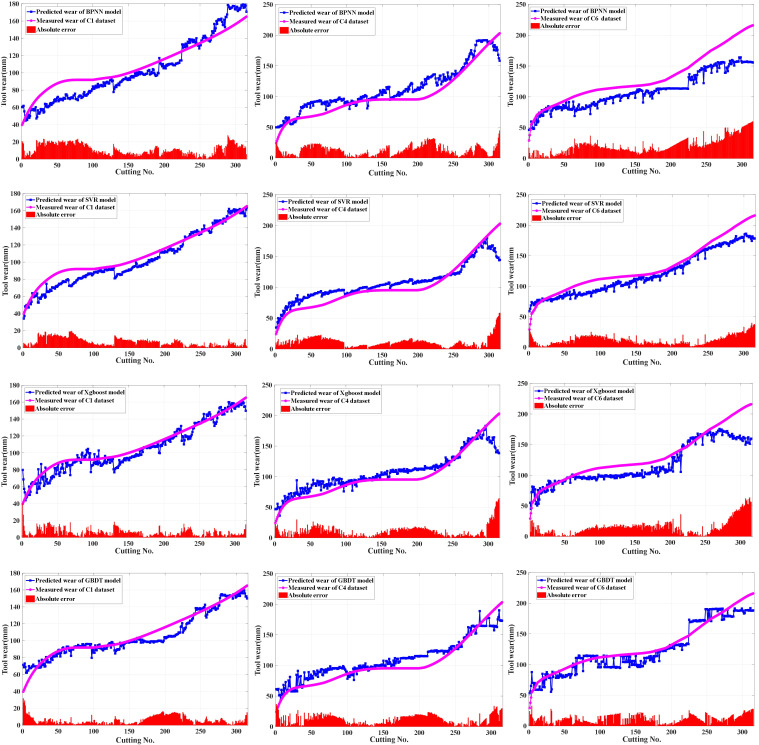
The predicted wear of the comparative prediction models for each datasets.

**Figure 6:**
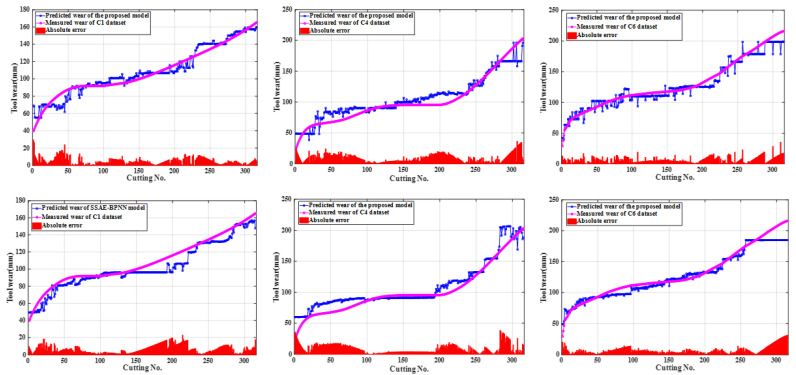
The predicted wear of the proposed method and single SSAE-BPNN model for each datasets.

**Figure 7:**
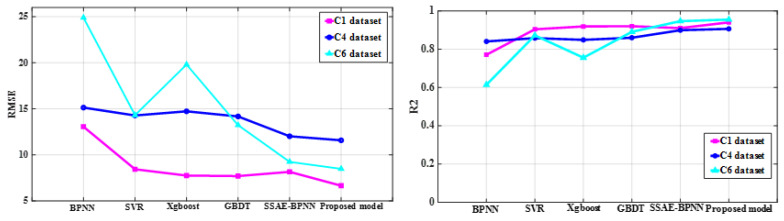
The comparison effect of the evaluation indexes for six different prediction models.

The proposed method applied deep learning models with different predictive performance as the primary learner. The hidden layer activation function of the SSAE model directly affected its feature learning ability and tool wear prediction performance. Sigmoid, hard_sigmoid, tanh, softsign, and sin activation functions were used for SSAE model training with the same optimal hyperparameter configuration for tool wear prediction, respectively. Considering that the output values of these functions fall within the range of −1 to 1 or 0 to 1, this ensured that the hidden layer outputs of the AE model remained within the same range as the input data combined with the construction process of the SSAE model and the normalization of the input data, which allowed the SSAE model to be trained layer-by-layer and perform deep feature extraction. The root mean square error (RMSE) of the predictive wear is listed in the Table 3 with C1 and C4 datasets as the training samples and C6 dataset as the test samples. The SSAE models configured with sigmoid, hard_sigmoid, softsign, and tanh activation functions were selected as the primary learners of the proposed model. Principal component analysis (PCA) was applied to compress and perform dimensionality reduction on the selected sensitive features. The BPNN, SVR, Xgboost, and GBDT regression models with the first five principal components as input and Bayesian optimization hyperparameters were established for predicting the changes in the wear of milling tool as comparative prediction models. The predicted wear of the above four constructed prediction models for the three datasets as test set are shown in Figure 5 with the model parameter configuration listed in Table 4. The GBDT regression model with Bayesian optimization hyperparameters showed better predictive performance compared to other shallow machine learning regression models, which was utilized as the secondary learner of the proposed model. Four primary learners divided the training set and testing set at 1:1 ratio and used the stacking learning strategy for ensemble learning to create the new sample datasets for training the secondary learners, when two datasets were chosen as the training sets with the leftover dataset used for testing from C1, C4, and C6. The predicted wear of the proposed method and single SSAE-BPNN model with sigmoid as the activation function is shown in Figure 6. The optimal hyperparameter configurations of the GBDT model for predicting the C1, C4, and C6 datasets are listed in Table 5. RMSE and determination coefficient (R2) were calculated as evaluation indexes for the performance of predicting tool wear. The calculated evaluation index values of the proposed method and above five comparative predictive models are presented in Table 6 and the comparison effect is shown in Figure 7.

All model algorithms were constructed and trained through the Python programming language utilizing the TensorFlow 1.80 and Sklearn 0.24 libraries. The selection of the model hyperparameters was determined by using the Hyperopt library and the Bayesian optimization method. Simultaneously, the proposed tool wear prediction model was completed through offline training, enabling it to meet the real-time requirements of practical applications.

## 5. Conclusions

In this paper, a new SSAE-based integrated model was constructed to predict tool wear. By using correlation analysis for sensitive feature selection, multi-sensor feature fusion technology facilitated the compression of and dimensionality reduction in multi-sensor signals. A layer-by-layer pretraining mechanism was applied in the SSAE model to generate deep fusion features in the top hidden layer with BPNN as the regression layer to establish the deep neural network model, which was trained through the weight transfer training strategy with the multi-sensor sensitive features as input for tool wear prediction. In comparison to the principal component components derived from multi-sensor feature combinations using PCA and input into BPNN, SVR, Xgboost, and GBDT models for tool wear prediction, the SSAE-BPNN model showed better predictive performance. By using sigmoid, hard_sigmoid, softsign, and tanh as hidden layer activation functions, the SSAE models were trained as the deep learning models with different prediction performance from data samples. The intergrated deep learning model used the stacking learning strategy with the above four trained SSAE models as primary learners and GBDT model with Bayesian optimization hyperparameters as secondary learner. Compared with the prediction models based on shallow machine learning techniques and the single SSAE-BPNN model, the proposed model exhibited superior prediction performance with achieving the lowest RMSE and highest R2, which further reduced prediction error and improved the stability of tool wear prediction with the R2 for the predictions of the C1, C4, and C6 datasets all greater than 0.9.

## Figures and Tables

**Table 1:** The experimental conditions in high-speed milling process.

Experimental Conditions	Parameters
CNC machine tool	Roders Tech RFM 760
Workpiece material	Stainless steel (HRC52)
Spindle speed	10,400 RPM
Feed rate	1555 mm/min
radial depth of cut	0.125 mm
axial depth of cut	0.2 mm
Sampling frequency	50 kHz

**Table 2:** The RMSE of SSAE models prediction results with different network parameters.

Correlation Coefficient	Neuron Nodes of SSAE	Neuron Nodes of BPNN	RMSE	Parameters
0.6	118-88-36-15	15-30-1	10.25	β = 0.2765, ρ = 0.8019
0.7	98-68-50-19	19-30-1	10.6	β = 0.9511, ρ = 0.8962
0.8	58-43-25-15	15-30-1	9.25	β = 0.4377, ρ = 0.7854

**Table 3:** The RMSE of SSAE models prediction results with different activation functions.

Evaluation Index	Activation Function				
	Sigmoid	Hard_Sigmoid	Tanh	Softsign	Sin
RMSE	9.25	9.56	29.46	19.39	47.36

**Table 4:** The parameter configuration of BPNN, SVR, Xgboost, and GBDT models.

Models	Nodes of Network	Activation Function	Learning_Rate	Hyperparameters
PCA + BPNN	5-10-1	Sigmoid	0.010	Epochs = 1000, Batch_size = 15
PCA + SVR	/	RBF	/	Gamma = 0.075, C = 643
PCA + Xgboost	/	/	0.012	Min_child_weight = 2, Colsample_bytree = 0.53, Max_depth = 1, N_estimtors = 400, Subsample = 0.31
PCA + GBDT	/		0.015	Max_depth = 3, Max_features = 4, N_estimtors = 130

**Table 5:** The parameter configuration of GBDT model for predicting C1, C4 and C6 datasets.

Datasets	Hyperparameters of GBDT			
	Learning_Rate	Max_Depth	Max_Features	N_Estimators
C1	0.53	12	1	400
C4	0.66	5	2	330
C6	0.86	9	2	320

**Table 6:** The calculated RMSE and R2 of the proposed model and comparative prediction models.

Model	C1		C4		C6	
	RMSE	R2	RMSE	R2	RMSE	R2
PCA + BPNN	13.0612	0.7712	15.1367	0.8407	24.8990	0.6141
PCA + SVR	8.4447	0.9044	14.2829	0.8582	14.3160	0.8724
PCA + Xgboost	7.7569	0.9193	14.7338	0.8491	19.8071	0.7558
PCA + GBDT	7.7035	0.9204	14.1754	0.8603	13.2319	0.8910
SSAE-BPNN	8.1644	0.9106	12.0244	0.8995	9.2474	0.9468
Proposed model	6.6576	0.9406	11.5857	0.9067	8.4880	0.9552

## Data Availability

The data that support the findings of this study are openly available in a public repository at https://pan.baidu.com/s/17GbX52SlPScsv0G7fDp5dQ#list/path=%2F (accessed on 6 April 2025).

## Abbreviations

The following abbreviations are used in this manuscript:

CNC	Computerized numerical control
SSAE	Stacked sparse autoencoder
BPNN	Backpropagation neural network
GBDT	Gradient boosting decision tree
PHM	Prognostics health management
SVM	Support vector machine
LSTM	Long-short term memory
ANN	Artificial neural network
SVR	Support vector regression
GPR	Gaussian process regression
AE	Autoencoder
RMSE	Root mean square error
PCA	Principal component analysis
R2	Determination coefficient
